# A high abundance of *Firmicutes* in the intestine of chinese mitten crabs (*Eriocheir sinensis*) cultured in an alkaline region

**DOI:** 10.1186/s13568-021-01301-w

**Published:** 2021-10-24

**Authors:** Weibing Guan, Kui Li, Shuang Zhao, Kejun Li

**Affiliations:** College of Marine Ecology and Environment, Shanghai Ocean University, Shanghai, China

**Keywords:** Bacterial diversity, Chinese mitten crab, Intestinal bacteria, rice–crab, Rice field

## Abstract

The Chinese mitten crab (*Eriocheir sinensis*) is a popular aquaculture product in East Asia, especially in China. In the last decade, rice–crab co-culture has rapidly expanded in China. Under this model, crabs are raised in rice fields instead of in traditional aquaculture ponds. In this study, we cultured two varieties of Chinese mitten crabs (Changjiang and Liaohe) in an alkaline region in northwest China and used Illumina MiSeq sequencing to compare the intestinal bacterial alpha diversity and community structure between traditional and co-culture aquaculture models, between two crab varieties, and between female and male crabs. Significant variations in intestinal bacterial communities were found between crab varieties and between female and male crabs but not between aquaculture models. These results show that rice–crab co-culture operations did not obviously impact the crab intestinal bacterial community compared with traditional pond aquaculture. *Firmicutes* was the most abundant bacterial phylum in the crab intestines (78%, relative abundance). Three dominant operational taxonomic units (OTUs) represented 73.2% of *Firmicutes* sequences and 56.8% of all sequences. A dominant OTU assigned as *Firmicutes* that was negatively correlated with crab body length, width, and weight was found in the source water for the experimental area. The results of this study suggest that the aquaculture of Chinese mitten crabs in alkaline regions requires more study to improve cultivation techniques.

**Supplementary Information:**

The online version contains supplementary material available at 10.1186/s13568-021-01301-w.

## Introduction

The Chinese mitten crab (*Eriocheir sinensis*) is a popular aquaculture product in East Asia. Traditionally, this crab is farmed in aquaculture ponds or natural lakes, but culture in rice fields has been encouraged in the last decade. rice–crab co-culture is an ecological breeding model which is environmentally sustainable and can have economic benefits (Zhao et al. 2020). This model is an integration of crop farming and crab aquaculture, thus researchers have studied the potential negative effects on aquaculture animals of fertilizers and pesticides used for plants and feed supplements used for animals (Li et al. 2021; Song et al. 2019). In alkaline regions in northwest China, rice–crab co-culture is being developed, but the impacts of alkalinity on Chinese mitten crab aquaculture remains unclear.

Intestinal bacteria are important for digestion and immunity in host animals. Intestinal bacteria have gained increasing attention in recent decades, and their biodiversity and community structure in different host species and under different conditions have been widely studied. A number of studies have shown that many factors affect the intestinal bacteria in Chinese mitten crabs (Ding et al. 2017; Dong et al. 2018). For example, Wang et al. (2019) reported that the gut microbiota diversity index in crabs decreased continuously with age and the effect of environmental microorganisms on the intestinal microbes gradually decreased as culture time increased. The microbial assemblages in the same tissues share a greater similarity than those from crabs of different sex and those that are fed different diets (Ma et al. 2021). Glyphosate, one of the most widely used pesticides, significantly decreased the diversity of crab gut microbiota while significantly increasing the taxonomic richness of *Bacteroidetes* and *Proteobacteria* (Yang et al. 2019). Ammonia nitrogen significantly decreased the relative abundance of *Bacteroidetes* in the crab gut (Yang et al. 2021).

The three varieties of Chinese mitten crabs in China are the Changjiang, Liaohe, and Oujiang, which originated from Changjiang, Liaohe, and Oujiang rivers respectively. The Changjiang variety is the representative Chinese mitten crab, with fast growth and a shiny carapace, and most studies of Chinese mitten crabs have focused on this variety. The Liaohe variety is mostly cultured in north China, and it is characterized by a thick carapace and better cold tolerance. The Oujiang variety is relatively small and rarely farmed. In addition to crab size, the degree of gonad and hepatopancreas development is an important factor in determining price, and female crabs are usually more popular with consumers.

In this study, we compared the intestinal bacterial present in Chinese mitten crabs (Changjiang and Liaohe, female and male) farmed in aquaculture ponds and rice fields to determine the impact of the co-culture on the intestinal bacteria. Results of this study provide a scientific basis for the development of co-culture technology, which is rapidly expanding in China.

## Materials and methods

### Experimental area

One established aquaculture-agriculture farm (106.36222 E, 38.62176 N) in an alkaline region in northwest China was used for the purposes of this study. The inflow water for each pond came from the Yellow River via canals. The outflow water from each pond was collected and used as irrigation for the rice fields. Sometimes the rice fields were also irrigated using Yellow River water. The Changjiang and Liaohe varieties of the Chinese mitten crab were raised in rice fields and also in aquaculture ponds, with the latter serving as the control. Water depth was approximately 10 cm for rice fields and 2.0–2.5 m for aquaculture ponds. Along the edge of each rice field, a circular groove with depth of approximately 50 cm and width of 2 m was excavated to provide shelter for the crabs when the water was too shallow in the rice fields. Two crab varieties were introduced into separate fields and ponds in March and April of 2020 at a stocking density of 70 kg per hectare in rice fields and 200 kg in ponds, with approximately 11 and 18 g per individual for the Liaohe and Changjiang varieties, respectively, according to local aquaculture methods. Rice was planted in early May. After planting, the crabs were fed with artificial compound feed (H201, Shuangyu, Liaoning, China), with a pellet size of 1.6 mm, that contained 38% crude protein, 18% crude ash, 12% crude fiber, 4% crude fat, 18% crude ash, 1% total phosphorus, 1.8% lysine, and 13.5% water. The crabs were harvested in September.

### Environmental analysis of the experimental area

Four aquaculture ponds and five rice–crab fields were randomly chosen for analysis of water quality in the experimental area from July to September of 2020, once a month. From the central site of each pond or field, alkalinity, phosphate, ammonia nitrogen (ammonia–N), and nitrite nitrogen (nitrite–N) content were measured once a month following Chinese National Environmental Protection Standards. Additionally, pH, dissolved oxygen content, temperature, and salinity were measured in situ using water quality instruments (Pocket Pro+ and HQ40, Hach, Loveland, CO, USA).

For the environmental bacteria survey, three aquaculture ponds, three rice fields, and four sites in the canal system (between ponds and fields) were sampled twice with a 2-week interval and the source water in the water inlet for the farm was sampled three times with a one-week interval in August of 2020. The 500 ml of water collected for each sample were filtered using a 5 μm pore filter and then a 0.22 μm pore filter. The 0.22 μm pore filters were then enclosed in sterile centrifuge tubes and stored at − 80 °C for later analysis.

Soil from the central site of six rice fields with areas ranging from 1.9 to 4.3 hm^2^ was sampled once in August of 2020 from 5 cm below the mud surface using a tubular stainless steel soil sampler. The outer layer of soil from each sample was removed using a sterile lancet, and the core soil was well mixed. For each sample, a 1 g aliquot of the core soil was reserved for subsequent analysis. All samples were stored in sterile centrifuge tubes at − 80 °C until subsequent analysis. In total, 23 water samples and 6 soil samples were collected.

### Intestinal bacteria sample collection

Crabs from six areas in rice fields and two aquaculture ponds were used to assess the intestinal bacteria assemblage. In total, 80 intestinal bacteria samples were collected in August of 2020. Additional file 1: Table S1 provides details about the study area and the yields of crab and rice in 2020. In each pond and field, five female crabs and five male crabs were randomly chosen for intestinal content sample collection. The length, width, weight, and wet weight of the carapace, gonad, and hepatopancreas were measured for each crab (n = 80, Additional file 2: Table S2). A sterile surgical lancet and scissors were used to rapidly kill the crabs and remove the whole intestinal tract. Sterile tweezers were used to squeeze the intestinal contents into sterile 1.5-ml centrifuge tubes. The intestinal contents in the centrifuge tubes were stored at − 80 °C until subsequent analysis. All experiments involving animals were performed in accordance with protocols approved by the Animal Care and Use Committee of Shanghai Ocean University (Approval ID: SHOU-DW-2020-058).

### 16 S rRNA gene sequencing

Bacterial community genomic DNA was extracted using the E.Z.N.A.^®^ soil DNA Kit (Omega Bio-tek, Norcross, GA, USA). Using the primers 338 F and 806R (Srinivasan et al. 2012) with barcodes, the V3–V4 region of the bacterial 16 S rRNA gene was amplified using an ABI GeneAmp^®^ 9700 PCR thermocycler (ABI, Carlsbad, CA, USA). DNA amplification was unsuccessful for one sample (X37). PCR reactions were performed in triplicate. The PCR product was extracted from agarose gel, purified, and quantified. Purified amplicons from same sample were pooled in equal amounts molar and pair-end sequenced (2 × 300) on an Illumina MiSeq platform (Illumina, San Diego, CA, USA). In this study, operational taxonomic units (OTUs) with a 97% similarity cutoff were clustered using Uparse v. 7.1. (Edgar 2013). The taxonomy of each OTU representative sequence was analyzed against the Silva v 138 16 S rRNA database (Quast et al. 2013). Shannon and Simpson indexes were used to identify bacterial alpha diversity, and Good’s coverage was calculated to characterize the sequencing depth using Mothur v.1.30.2 (Schloss et al. 2009). Details about 16 S rRNA sequencing, raw read processing, and OTU clustering are provided in a previous paper (Bao et al. 2021). Representative sequences of the OTUs from different libraries were aligned using the BLAST tool (https://blast.ncbi.nlm.nih.gov/Blast.cgi). Sequences from 79 intestinal samples and 29 environmental samples were also pooled and clustered to detect some certain OTUs, using Uparse.

### Sequence submission and statistical analysis

The raw 16 S rRNA gene sequences obtained in this study have been submitted to the NCBI Sequence Read Archive database (https://submit.ncbi.nlm.nih.gov/subs/sra/) under the accession number SRP325796. Statistical analysis was carried out using R version 3.3.1 (The R Foundation, Vienna, Austria) and SPSS 18.0 (IBM, Armonk, NY, USA). Inter-group variations were detected using analysis of similarities (R vegan package). Significance levels of differences between sample groups were detected using Student’s t-test and Wilcox rank-sum test (R stats package). Venn diagram analysis (R VennDiagram package) was used to reveal the numbers of opportunistic and core bacterial OTUs. The correlation between intestinal bacteria and body parameters was evaluated using Spearman correlation heatmap analysis (R pheatmap package). Analysis of variance (SPSS) was performed to compare the measured parameters of crabs. The independent-sample t-test (SPSS) was used to detect significant differences of water quality parameters between the two aquaculture systems.

## Results

### Comparison of body parameters of Chinese mitten crabs

**Fig. 1:**
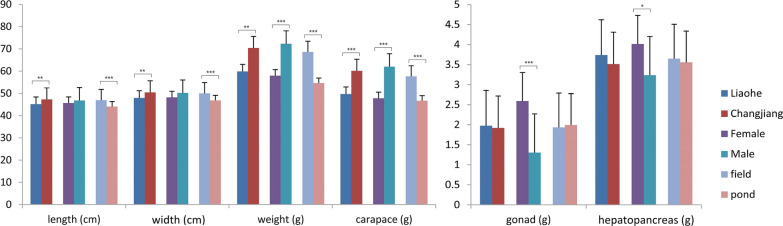
Body length, width, and weight and wet weight of the carapace, gonad, and hepatopancreas of the sampled Chinese mitten crabs cultured in rice fields and aquaculture ponds. Field and pond, crabs cultured in rice fields and aquaculture ponds; Liaohe and Changjiang, two varieties of Chinese mitten crab

Additional file 2: Table S2 lists the measured body parameters of the 80 crabs. The average body weight of the sampled crabs was 57.9 ± 12.9 g for the Liaohe variety and 72.3 ± 23.0 g for the Changjiang variety (Fig. 1). The Changjiang variety was 14.4 g heavier than the Liaohe variety, whereas the difference in weight was approximately 7 g heavier at the beginning of the experiment. Body length (P = 0.018), body width (P = 0.014), body weight (P = 0.017), and carapace weight (P = 0.006) were significantly higher in the Changjiang variety than in the Liaohe variety. Body weight (P = 0.001) and carapace weight (P < 0.001) were significantly higher in male crabs, but gonad weight (P < 0.001) and hepatopancreas weight (P = 0.024) were higher in female crabs. Length (P = 0.001), width (P = 0.001), body weight (P = 0.006), and carapace weight (P = 0.001) of rice field-raised crabs was significantly higher than those of pond-raised crabs. These results indicate that rice-field culture is beneficial to the growth of Chinese mitten crabs in alkaline regions.

### Comparison of intestinal bacteria in Chinese mitten crabs

Following Illumina MiSeq sequencing, 4.6 million valid sequences were obtained from 79 intestinal samples. After sub-sampling, 30,511 sequences from each sample were used in subsequent analysis. From these sub-sampled libraries, a total of 5056 OTUs were obtained. Good’s coverage values of all samples were greater than 98%, which means that the sequencing depth was sufficient to analyze the bacterial communities (Additional file 2: Table S2). The rarefaction curves also illustrated that more sequences would not substantially increase the number of OTUs (Additional file 6: Fig. S1). The results of Student’s *t*-test of Shannon and Simpson indexes revealed that there were no significant variations were found between crab varieties, between female and male crabs, and between pond-raised and rice field-raised crabs. Comparing bacterial communities between sample groups, the results of analysis of similarities at the OTU level showed that significant variations were found between crab varieties (R = 0.054, P = 0.002) and between female and male crab (R = 0.026, P = 0.04), but not between aquaculture ponds and rice fields (P = 0.39). Even with the same variety and sex, the variations between aquaculture ponds and rice fields were also not significant (P > 0.05). The low R values indicate that grouping has a low explanation degree for inter-sample differences. The results revealed that rice–crab co-culture did not obviously impact the intestinal bacteria in crabs.

### Intestinal bacteria in Chinese mitten crabs cultured in an alkaline region

**Fig. 2:**
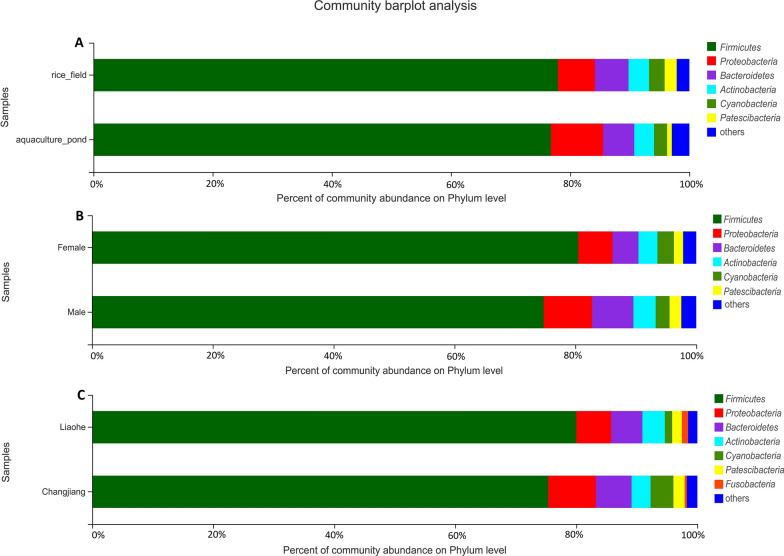
Bacterial community structure of the intestinal sample groups at the phylum level. Field and pond, intestinal bacteria in Chinese mitten crabs raised in the rice–crab co-culture system (n = 59) and aquaculture ponds (n = 20); female and male, female and male crabs (n = 40 and 39); Liaohe and Changjiang, two varieties of Chinese mitten crab (n = 39 and 40)

**Fig. 3:**
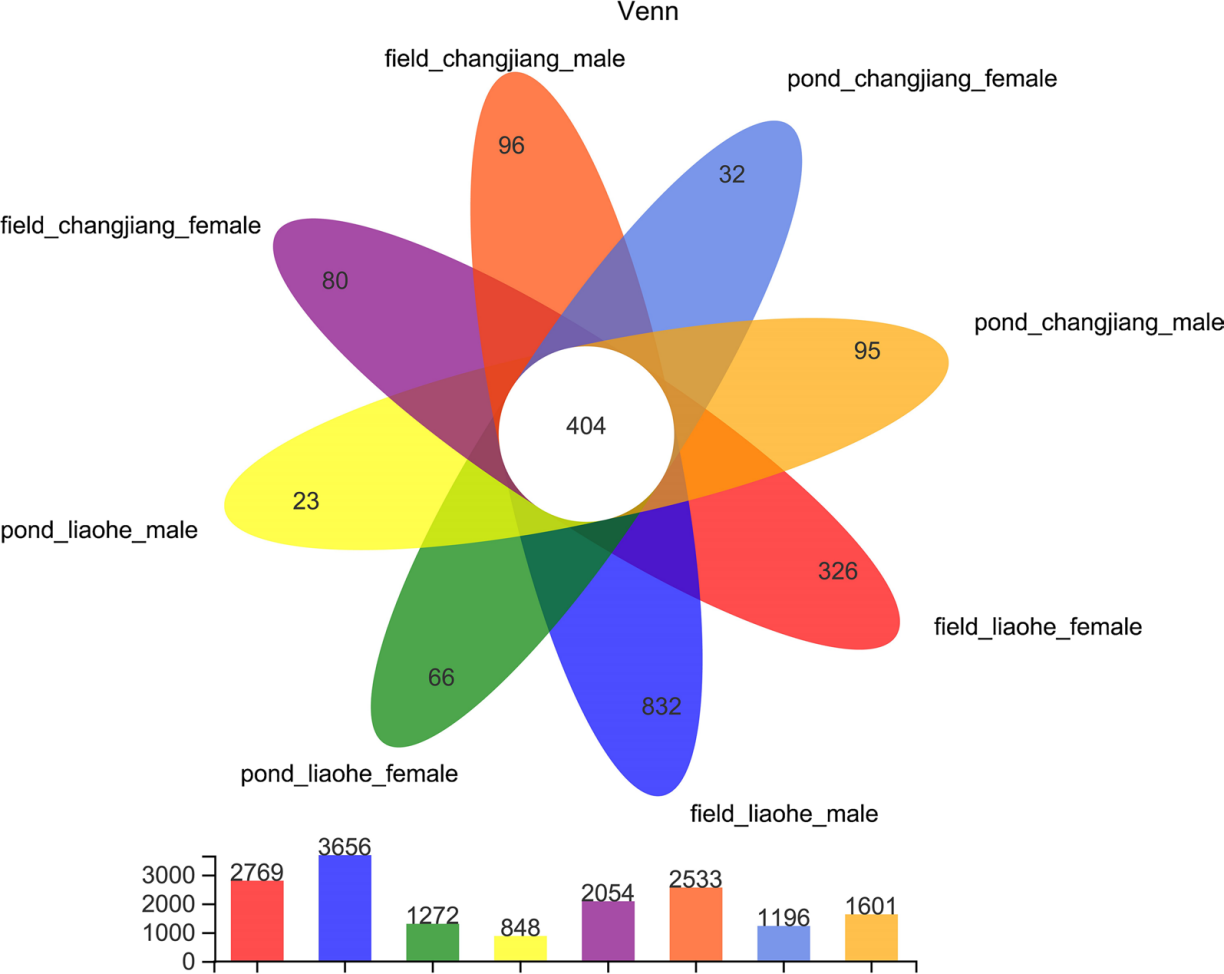
Venn diagram showing that core bacterial OTUs dominated the intestines of crabs, except for the Liaohe variety cultured in rice fields. The latter had more exclusive OTUs than crabs in the other sample groups. Field and pond, intestinal bacteria in Chinese mitten crabs raised in rice fields and aquaculture ponds; Liaohe and Changjiang, two varieties of Chinese mitten crab

**Fig. 4:**
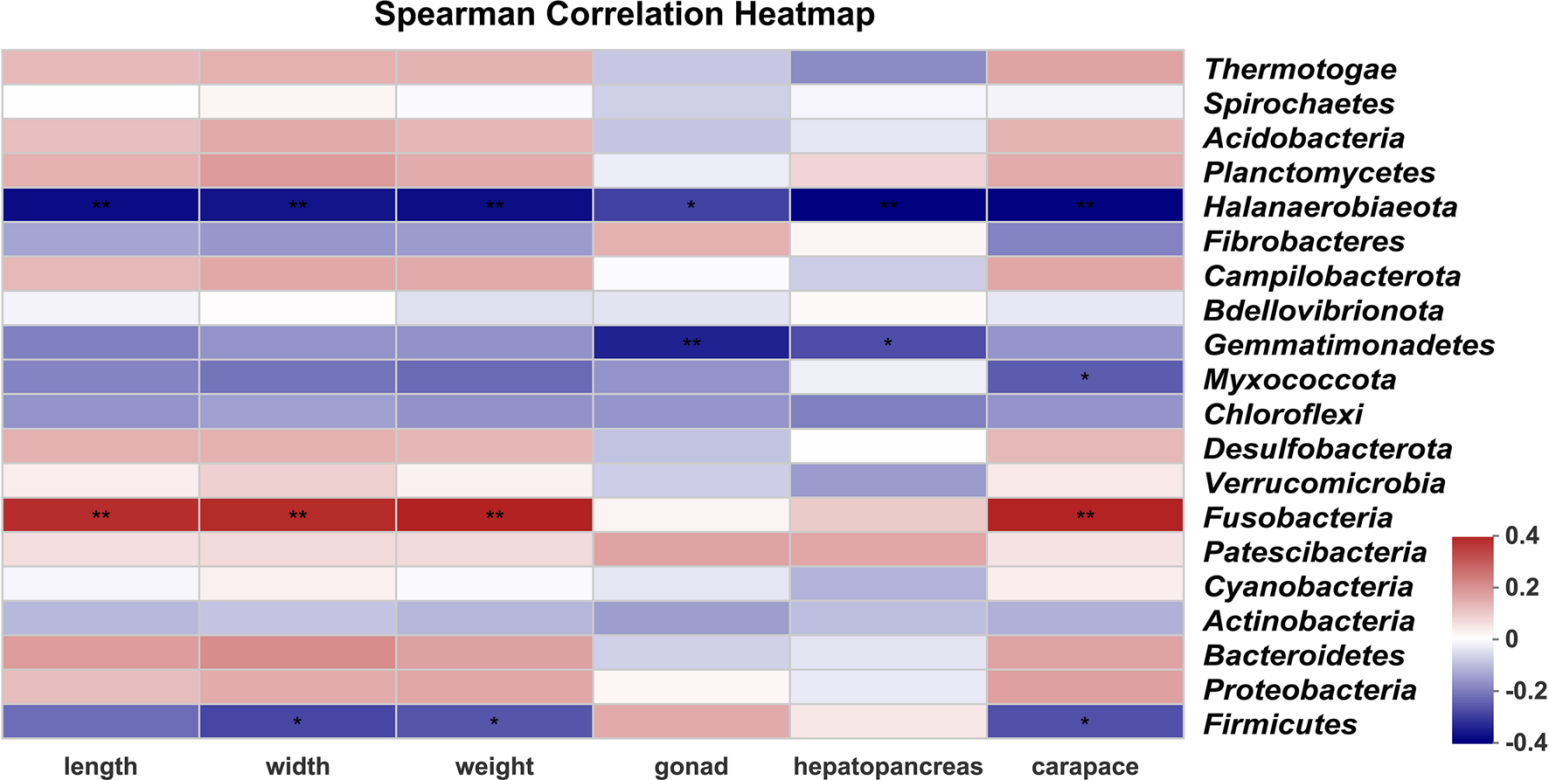
Spearman correlation heatmap showing the correlation between 20 dominant phyla and body parameters of sampled crabs. Length, width, and weight represent the body length, width, and weight of crabs; carapace, gonad, and hepatopancreas represent the wet weight of the carapace, gonad, and hepatopancreas. *Firmicutes*, the most dominant phylum, was negatively correlated with body width and weight of sampled crabs. Positive correlations are shown in red and negative correlations are shown in blue. * P < 0.05; ** P < 0.01

At the phylum level, *Firmicutes* represented approximately 78% of all analyzed sequences (Fig. 2), and *Proteobacteria*, *Bacteroidetes*, *Actinobacteria*, *Cyanobacteria*, and *Patescibacteria* represented more than 1% of sequences respectively. Results of the Wilcoxon rank-sum test revealed that of the 15 dominant phyla in intestinal samples, only *Verrucomicrobia* varied significantly between pond-raised and rice field-raised crabs. Further analyses showed that *Cyanobacteria*, *Myxococcota*, and *Fibrobacteres* varied significantly between the two crab varieties, but there were no differences between female and male crabs (Additional file 7: Fig. S2). The Venn diagram at the OTU level also revealed that core bacterial OTUs dominated the intestines of crabs, except for in the Liaohe variety cultured in rice fields (Fig. 3). Of the Liaohe crabs cultured in rice fields, male crabs had 832 exclusive OTUs and female crabs had 326 exclusive OTUs. In contrast, there were not more than 96 exclusive OTUs in any single other sample group. The results of Spearman correlation analysis showed that the dominant phylum, *Firmicutes*, was negatively related to body width and weight of crabs and wet weight of crab carapaces (Fig. 4).

We retrieved the OTUs assigned as *Firmicutes* from the 79 libraries and found that the most dominant OTUs were OTU4365, OTU3923, and OTU2976, which represented 32.4%, 21.4%, and 19.4% of *Firmicutes* sequences, respectively (Additional file 8: Fig. S3). Additionally, 56.8% of all analyzed sequences were assigned to these three *Firmicutes* OTUs. Additional file 3: Table S3 provides details about the representative sequences. Results of Spearman correlation analysis showed that OTU2976 was negatively related to body length, width, and weight of crabs and wet weight of crab carapaces (Additional file 9: Fig. S4).

### Water quality and environmental bacteria in the alkaline experimental area

The results of the independent-sample t-test showed that dissolved oxygen content differed significantly between the two aquaculture systems. The dissolved oxygen content was significantly higher (13.86 ± 0.83 mg l^−1^) in rice fields than in ponds (4.7 ± 3.12 mg l^−1^). The salinity and nitrite–N content in ponds were higher than that in rice fields, although the values in both settings were relatively low. In rice fields and ponds, the salinity content was 0.52 ± 0.13 ppt and 0.6 ± 0.03 ppt; the nitrite–N content was 0.001 ± 0.002 mg l^−1^ and 0.173 ± 0.105 mg l^−1^, respectively. The alkalinity was considerably high, 318 ± 45 mg l^−1^ and 307 ± 116 mg l^−1^ respectively (Additional file 4: Table S4).

**Fig. 5:**
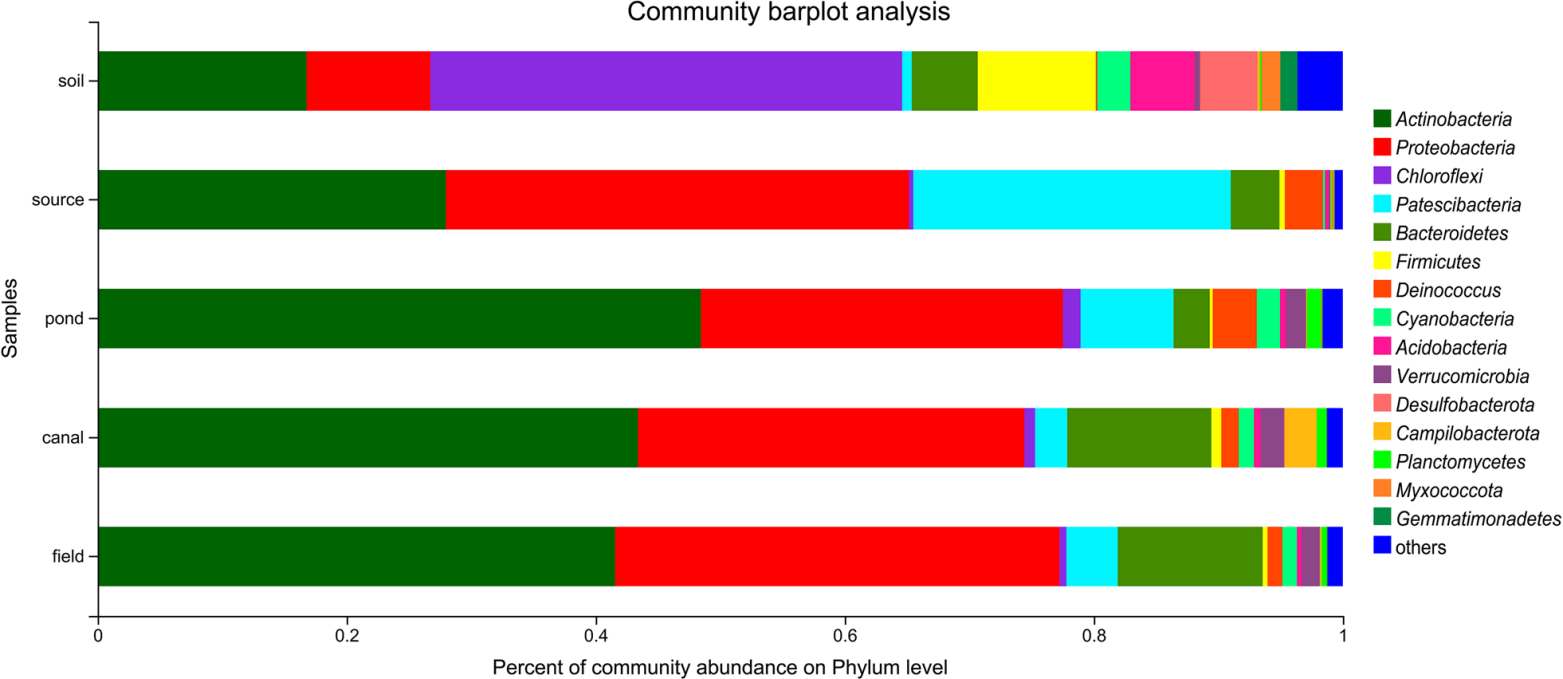
Bacterial community structure of five environmental sample groups at the phylum level. Soil, soil bacteria samples from six areas in rice fields; source, pond, and field, planktonic bacteria samples collected from the water inlet for the experimental area, from three aquaculture ponds, and from three areas in rice fields; canal, planktonic bacteria samples collected from the canal system between aquaculture and rice fields. A higher relative abundance of *Firmicutes* was found in the soil bacteria samples than in the water samples

After subsampling, 38,296 sequences for each collected environmental sample (23 water samples and 6 soil samples) were used for analysis. From the 29 subsampled libraries, 6618 OTUs were obtained. Good’s coverage values of all samples were > 97.6%, which showed that the sequencing depth was sufficient to analyze the bacterial communities (Additional file 5: Table S5). At the phylum level, *Actinobacteria*, *Proteobacteria*, and *Patescibacteria* dominated the water samples collected in the water inlet for the farm; *Actinobacteria* and *Proteobacteria* dominated the water samples collected in the farm; and *Chloroflexi*, *Actinobacteria*, *Proteobacteria*, and *Firmicutes* dominated the soil in the rice fields. *Firmicutes* represented approximately 2.5% and 9.9% of sequences from all 29 samples and from the soil samples respectively (Fig. 5).

Aligning representative sequences of the three dominant *Firmicutes* OTUs from the crab intestines with the OTU representative sequences found in the environment revealed one OTU from environmental samples with similarity of 97.2% with OTU2976. This environmental OTU represented five sequences from the water inlet and two sequences from soil and water in the farm. After sub-sampling and OTU clustering of Sequences from 79 intestinal samples and 29 environmental samples, 30,458 sequences from each sample were used in subsequent analysis and a total of 10,058 OTUs were obtained. Comparing the representative sequences, one OTU with a 100% similarity with OTU2976 represented more than 0.36 million of sequences in the intestinal samples and two sequences in water samples from the canal and inlet. This result suggests that the high abundance OTU2976 in crab intestines could have originated from the environment, especially the source water.

## Discussion

Many factors impact the growth of Chinese mitten crabs. Compared with aquaculture ponds, rice fields are shallow, and shallow water can lead to supersaturated dissolved oxygen via photosynthesis by algae (Long et al. 2020). In oxygen minimum zones, Seibel et al. (2018) found metabolic suppression in the pelagic crab, *Pleuroncodes planipes*. Jia et al. (2018) reported that reactive oxygen species sensitized hematopoietic progenitors in Chinese mitten crabs to differentiation and played a signaling role in the regulation of hematopoietic cell fate. Ambient salinity is another critical factor that affects crab culture. Salinity can change the lipid composition of adult Chinese mitten crabs after long-term salinity adaptation (Long et al. 2019), and Qi et al. (2020) described novel modulation of the physiological regulation mechanism in cultured Chinese mitten crabs in response to consistent salinity changes. Dietary structure and composition also significantly impact the growth, body composition, immunity, and gene expression of Chinese mitten crab (Wei et al. 2019a; Lu et al. 2019). However, little is known about the effect of alkalinity on intestinal bacteria in Chinese mitten crabs. Because aquaculture of Chinese mitten crabs in alkaline regions can improve land utilization efficiency and provide a local supply of this aquatic product, it is crucial to improve our understanding of the impacts of alkalinity on the growth of this species.

Intestinal bacteria in Chinese mitten crabs are influenced by many factors, including drugs and feed additives (Yang et al. 2019) and pesticides (Hong et al. 2020). Studies have shown that the gut microbiome community is significantly affected by habitat (Chen et al. 2021) and developmental stage (Wang et al. 2019). In this study, we found that rice–crab co-culture did not obviously impact the intestinal bacteria in crabs relative to traditional pond culture. In contrast, Cheng et al. (2017) found obvious differences in the intestinal bacteria community in Chinese mitten crabs (Changjiang) between small rice–crab co-culture simulative systems with and without rice planting. It is possible that the difference was due to the use of aquaculture water with high alkalinity in the present study. Some bacteria have strategies to adapt to the acidity or alkalinity of the cytoplasm (Nyanga-Koumou et al. 2012). For example, Zhao et al. (2010) found that different functional microbial populations responded well to the alkalinity changes in a sulfidogenic bioreactor. In several crater lakes along an alkalinity gradient, microbial community structure was correlated with lake physicochemical parameters, notably alkalinity (Iniesto et al. 2021). Changes in the intestinal bacteria community structure reflect the overall health status of host animals, and community structure stability is very important (Xiong et al. 2015). In the current study, we found no significant impacts of rice–crab co-culture operations on the intestinal bacteria.

*Firmicutes* represented 78% of analyzed sequences in the current study. Previous studies reported that other phyla, such as *Proteobacteria*, *Acidobacteria*, and *Bacteroidetes*, dominated the intestinal assemblage of Chinese mitten crabs (Shi et al. 2019; Su et al. 2020), fiddler crabs (Cuellar-Gempeler and Leibold 2018, 2019), mud crabs (Wei et al. 2019b; Lin et al. 2020), and vent crabs (Zhang et al. 2017). We also found that *Proteobacteria* and *Bacteroidetes* dominated wild and pond-raised Chinese mitten crabs (Li et al. 2007). In other studies, *Tenericutes* was the most dominant phylum in Chinese mitten crabs (Chen et al. 2015; Zhang et al. 2016, Yang et al. 2019). Ding et al. (2017) reported a high relative abundance of *Firmicutes* (34.9–35.3%) in Chinese mitten crabs during various stages of white spot syndrome virus infection. To the best of our knowledge, the high proportion of *Firmicutes* in Chinese mitten crab intestines found in the present study has not been previously reported. Most studies of intestinal bacteria in Chinese mitten crabs have focused on crabs cultured in low-alkaline regions such as in eastern China, whereas the crabs involved in this study were cultured in an alkaline region in the northwest China.

The normal human gut microbiome consists of two major phyla, Bacteroidetes and *Firmicutes* (Jandhyala et al. 2015). Obesity has been associated with an altered gut microbiome characterized by elevated levels of *Firmicutes* and depleted levels of *Bacteroidetes* (Koliada et al. 2017; Riva et al. 2017). Data also indicate that the composition of human intestinal microbiota at the level of major microbial phyla significantly differs across age groups, and in both sexes the *Firmicutes*/*Bacteroidetes* ratio tends to increase with age (Vaiserman et al. 2020). However, we found that *Firmicutes* abundance was negatively related to Chinese mitten crab weight in the present study. In particular, OTU 2976 dominated the crab intestine microbiome and was negatively correlated with crab weight. The high proportion of *Firmicutes* in the crab intestine and the effect of *Firmicutes* on crabs in alkaline regions merit further study.

Although rice–crab co-culture is rapidly expanding in China, additional research is required to further develop this technology. In the current study, we analyzed intestinal bacteria in Chinese mitten crabs raised in an alkaline region and found a high abundance of *Firmicutes*. In addition, a dominant *Firmicutes* OTU that was negatively correlated with crab weight was also found in the source water for the experimental area. These results suggest that more studies of intestinal bacteria in Chinese mitten crabs cultured in different regions are needed to improve cultivation techniques.

## Supplementary Information

**Additional file 1: Table S1.** Information about the two aquaculture ponds and six rice field areas (rice–crab co-culture system) and aquaculture operations.

**Additional file 2: Table S2**. Information about the intestinal samples and alpha diversity indexes.

**Additional file 3: Table S3**. Representative sequences of the three dominant *Firmicutes* OTUs with high abundances in crab intestines.

**Additional file 4: Table S4.** Water environment parameters in the experimental area.

**Additional file 5: Table S5**. Information about the environmental bacteria samples and alpha diversity indexes.

**Additional file 6: Figure S1**. The rarefaction curves illustrated that the sequencing depth was sufficient to analyze the bacterial communities. Field and pond, crabs cultured in rice fields and aquaculture ponds; Liaohe and Changjiang, two varieties of Chinese mitten crab; female and male, female and male crabs.

**Additional file 7: Figure S2**. Of the 15 dominant phyla in intestinal samples, just one phylum varied significantly between the two aquaculture models (A), no differences between female and male crabs were detected (B), and 3 phyla varied significantly between the two crab varieties (C).

**Additional file 8: Figure S3**. The community heatmap shows 50 dominant *Firmicutes* OTUs and their relative abundances. The most dominant OTUs were OTU2976, OTU3923, and OTU4365. High relative abundances are shown in red and low relative abundances are shown in blue.

**Additional file 9: Figure S4**. The Spearman correlation heatmap shows the correlation between the 50 dominant *Firmicutes* OTUs and body parameters of sampled crabs. OTU2976 was negatively related to body length, width, and weight of sampled crabs. Positive correlations are shown in red and negative correlations are shown in blue. * P < 0.05; ** P < 0.01; *** P < 0.001.

## Data Availability

The data that support the findings of this study are available in the supplementary material of this article. The raw gene sequences obtained in this study were submitted to the NCBI Sequence Read Archive database (https://submit.ncbi.nlm.nih.gov/subs/sra/) under the accession number SRP325796.
