# Supplementary figures and images for: A high abundance of Firmicutes in the intestine of chinese mitten crabs (Eriocheir sinensis) cultured in an alkaline region
Source: AMB Express. 2021 Oct 24;11:141. doi: 10.1186/s13568-021-01301-w (PMC8542526; doi:10.1186/s13568-021-01301-w)

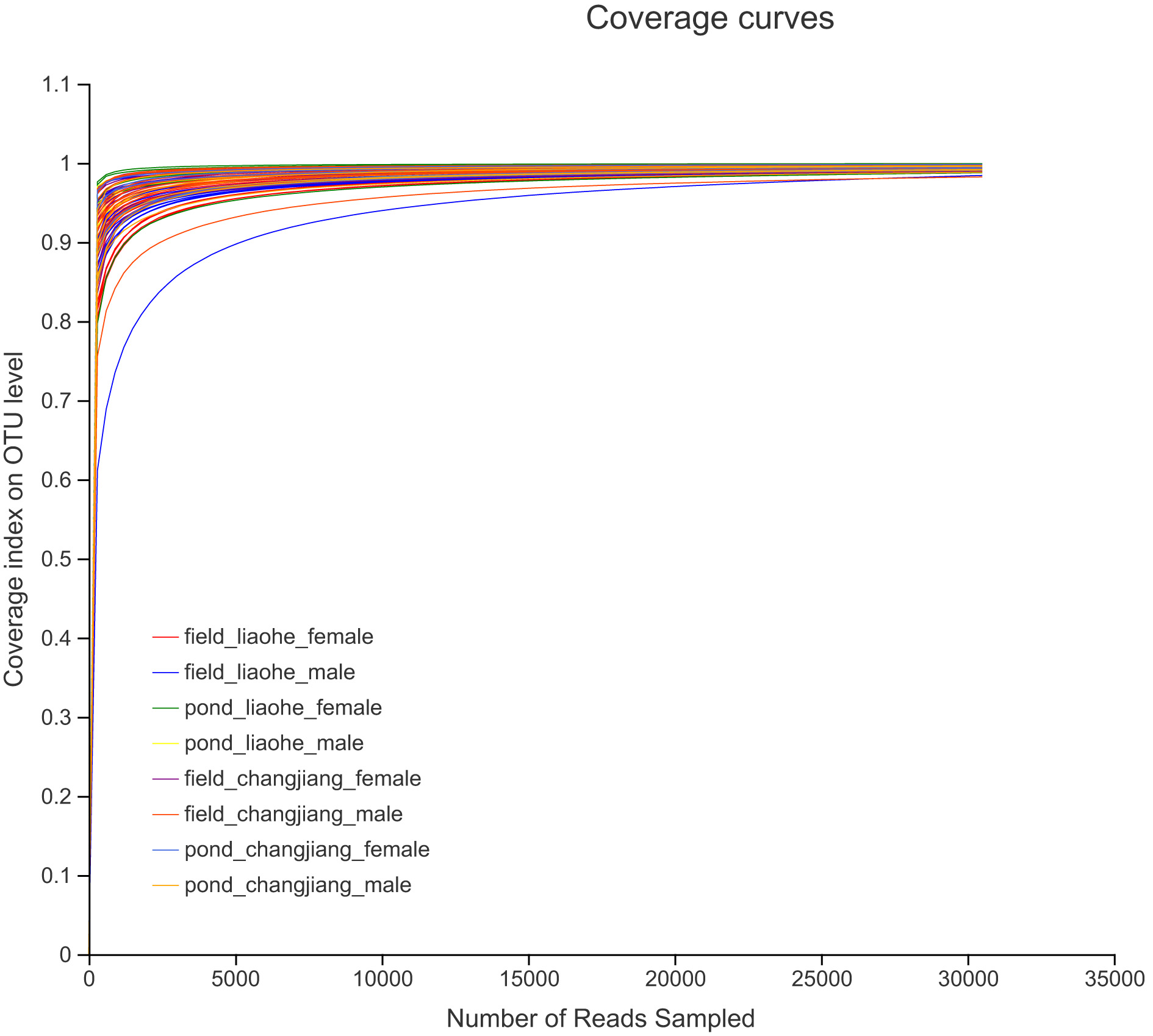

Supplement: Supplementary file 6 — Additional file 6: Figure S1. The rarefaction curves illustrated that the sequencing depth was sufficient to analyze the bacterial communities. Field and pond, crabs cultured in rice fields and aquaculture ponds; Liaohe and Changjiang, two varieties of Chinese mitten crab; female and male, female and male crabs. [file 13568_2021_1301_MOESM6_ESM.jpg]

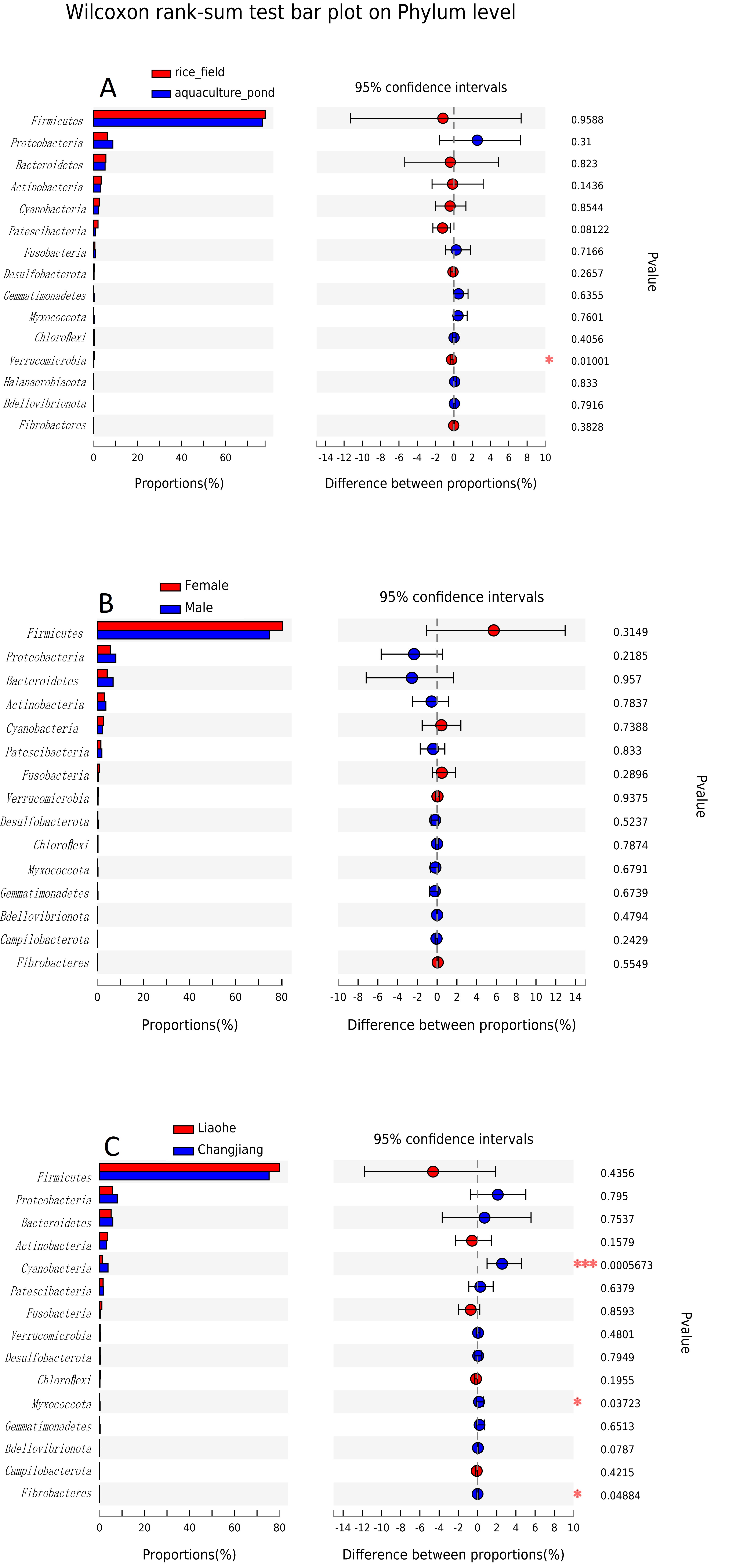

Supplement: Supplementary file 7 — Additional file 7: Figure S2. Of the 15 dominant phyla in intestinal samples, just one phylum varied significantly between the two aquaculture models (A), no differences between female and male crabs were detected (B), and 3 phyla varied significantly between the two crab varieties (C). [file 13568_2021_1301_MOESM7_ESM.jpg]

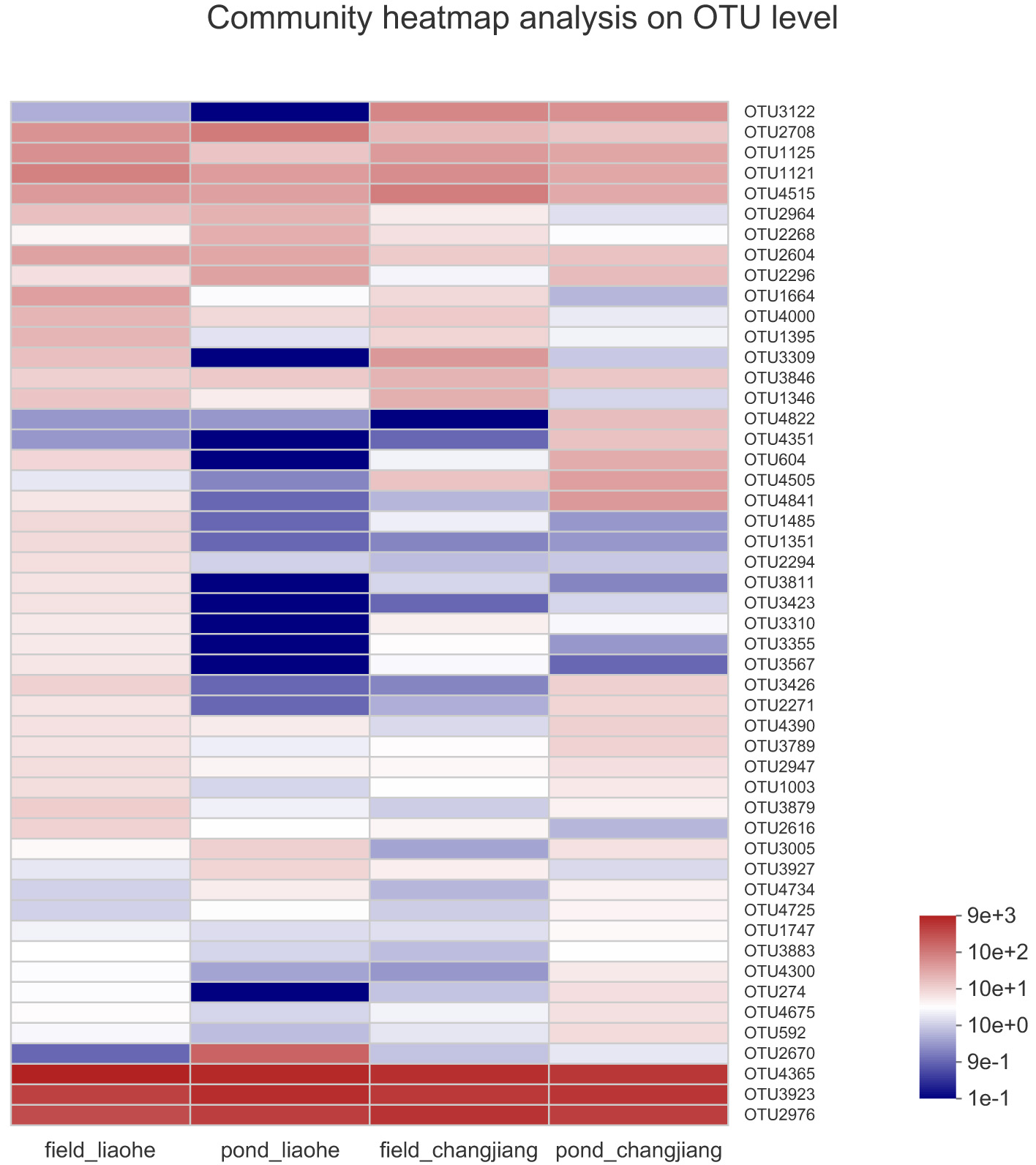

Supplement: Supplementary file 8 — Additional file 8: Figure S3. The community heatmap shows 50 dominant Firmicutes OTUs and their relative abundances. The most dominant OTUs were OTU2976, OTU3923, and OTU4365. High relative abundances are shown in red and low relative abundances are shown in blue. [file 13568_2021_1301_MOESM8_ESM.jpg]

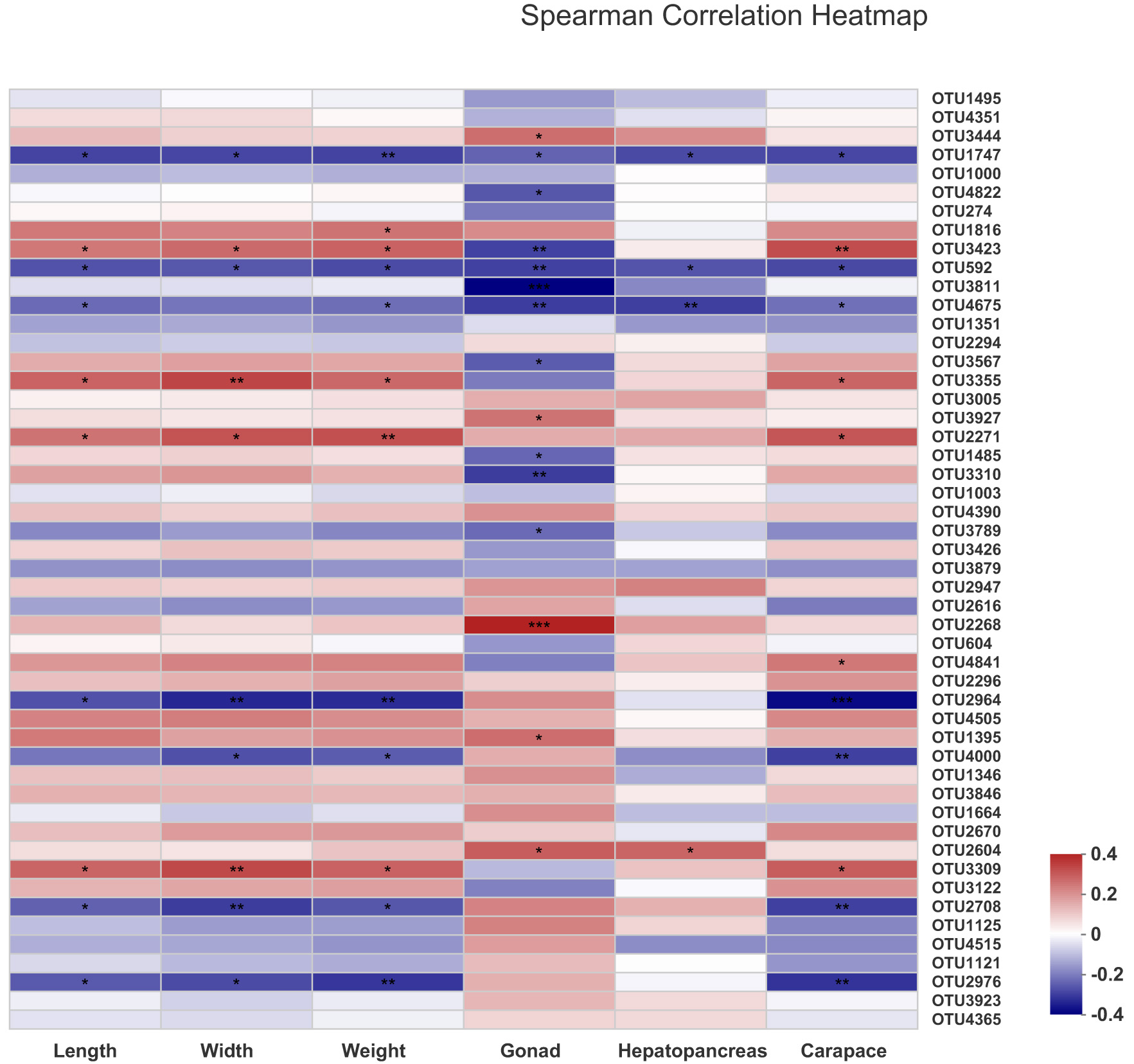

Supplement: Supplementary file 9 — Additional file 9: Figure S4. The Spearman correlation heatmap shows the correlation between the 50 dominant Firmicutes OTUs and body parameters of sampled crabs. OTU2976 was negatively related to body length, width, and weight of sampled crabs. Positive correlations are shown in red and negative correlations are shown in blue. * P < 0.05; ** P < 0.01; *** P < 0.001. [file 13568_2021_1301_MOESM9_ESM.jpg]
